# Y chromosome haplogroups and prostate cancer in populations of European and Ashkenazi Jewish ancestry

**DOI:** 10.1007/s00439-012-1139-5

**Published:** 2012-01-24

**Authors:** Zhaoming Wang, Hemang Parikh, Jinping Jia, Timothy Myers, Meredith Yeager, Kevin B. Jacobs, Amy Hutchinson, Laurie Burdett, Arpita Ghosh, Michael J. Thun, Susan M. Gapstur, W. Ryan Diver, Jarmo Virtamo, Demetrius Albanes, Geraldine Cancel-Tassin, Antoine Valeri, Olivier Cussenot, Kenneth Offit, Ed Giovannucci, Jing Ma, Meir J. Stampfer, J. Michael Gaziano, David J. Hunter, Ana Dutra-Clarke, Tomas Kirchhoff, Michael Alavanja, Laura B. Freeman, Stella Koutros, Robert Hoover, Sonja I. Berndt, Richard B. Hayes, Ilir Agalliu, Robert D. Burk, Sholom Wacholder, Gilles Thomas, Laufey Amundadottir

**Affiliations:** 1 Division of Cancer Epidemiology and Genetics, National Cancer Institute, National Institutes of Health, Bethesda, MD 20892 USA; 2 Core Genotyping Facility, SAIC-Frederick, Inc., NCI-Frederick, Frederick, MD 21702 USA; 3 Laboratory of Translational Genomics, Division of Cancer Epidemiology and Genetics, National Cancer Institute, National Institutes of Health, Bethesda, MD 20877 USA; 4 Epidemiology Research Program, American Cancer Society, Atlanta, GA 30303 USA; 5 Department of Chronic Disease Prevention, National Institute for Health and Welfare, 00300 Helsinki, Finland; 6 Centre de Recherche pour les Pathologies Prostatiques (CeRePP), Hôpital Tenon, Assistance Publique-Hôpitaux de Paris, 75020 Paris, France; 7 Clinical Genetics Service, Department of Medicine, Memorial Sloan-Kettering Cancer Center, Box 192, 1275 York Avenue, New York, NY 10065 USA; 8 Channing Laboratory, Division of Preventive Medicine, Department of Medicine, Brigham and Women’s Hospital, Harvard Medical School, Boston, MA 02115 USA; 9 Program in Molecular and Genetic Epidemiology, Department of Epidemiology, Harvard School of Public Health, Boston, MA 02115 USA; 10 Division of Epidemiology, Department of Environmental Medicine, New York University School of Medicine, New York, NY 10016 USA; 11 Department of Epidemiology and Population Health, Albert Einstein College of Medicine, Bronx, NewYork, NY 10461 USA; 15 Department of Pediatrics, Albert Einstein College of Medicine, Bronx, NewYork, NY 10461 USA; 16 Department of Microbiology & Immunology, Albert Einstein College of Medicine, Bronx, NewYork, NY 10461 USA; 17 Department of Obstetrics, Gynecology and Women’s Health, Albert Einstein College of Medicine, Bronx, NewYork, NY 10461 USA; 12 Synergie-Lyon-Cancer, Universite Lyon 1, Centre Leon Berard, 69373 Lyon Cedex 08, France; 13 Laboratory of Translational Genomics, Division of Cancer Epidemiology and Genetics, National Cancer Institute, National Institutes of Health, Gaithersburg, MD 20877 USA

## Abstract

Genetic variation on the Y chromosome has not been convincingly implicated in prostate cancer risk. To comprehensively analyze the role of inherited Y chromosome variation in prostate cancer risk in individuals of European ancestry, we genotyped 34 binary Y chromosome markers in 3,995 prostate cancer cases and 3,815 control subjects drawn from four studies. In this set, we identified nominally significant association between a rare haplogroup, E1b1b1c, and prostate cancer in stage I (*P* = 0.012, OR = 0.51; 95% confidence interval 0.30–0.87). Population substructure of E1b1b1c carriers suggested Ashkenazi Jewish ancestry, prompting a replication phase in individuals of both European and Ashkenazi Jewish ancestry. The association was not significant for prostate cancer overall in studies of either Ashkenazi Jewish (1,686 cases and 1,597 control subjects) or European (686 cases and 734 control subjects) ancestry (*P*_meta_ = 0.078), but a meta-analysis of stage I and II studies revealed a nominally significant association with prostate cancer risk (*P*_meta_ = 0.010, OR = 0.77; 95% confidence interval 0.62–0.94). Comparing haplogroup frequencies between studies, we noted strong similarities between those conducted in the US and France, in which the majority of men carried R1 haplogroups, resembling Northwestern European populations. On the other hand, Finns had a remarkably different haplogroup distribution with a preponderance of N1c and I1 haplogroups. In summary, our results suggest that inherited Y chromosome variation plays a limited role in prostate cancer etiology in European populations but warrant follow-up in additional large and well characterized studies of multiple ethnic backgrounds.

**Electronic supplementary material:**

The online version of this article (doi:10.1007/s00439-012-1139-5) contains supplementary material, which is available to authorized users.

## Introduction

Family and twin studies have shown that prostate cancer has a clear heritable component which may be among the highest of all cancer types (Amundadottir et al. 2004; Lichtenstein et al. 2000), Over the last few years, genome wide association studies (GWAS) have successfully identified germline variants conferring risks of prostate cancer at over 45 loci (Amundadottir et al. 2006; Chung and Chanock 2011; Eeles et al. 2008, 2009; Gudmundsson et al. 2007a, b, 2008, 2009; Haiman et al. 2007; Kote-Jarai et al. 2011; Schumacher et al. 2011; Takata et al. 2010; Thomas et al. 2008; Yeager et al. 2007, 2009). These studies have not implicated variants on the Y chromosome in the risk of prostate cancer, possibly due to the fact that very few Y chromosome SNPs have been included on most genotyping chips used to date. Several groups have specifically investigated the role of Y chromosome haplogroups in prostate cancer risk. Many of these studies are inconclusive due to the small number of samples and/or markers used. One of the larger studies was conducted within the multi-ethnic cohort (MEC) using samples from prostate cancer cases and control subjects drawn from four ethnic groups. Of the 41 haplogroups observed, one was significantly associated with prostate cancer in Japanese men (Paracchini et al. 2003) but this association was not replicated in a separate study from Korea (Kim et al. 2007). No association was seen between Y haplogroups and prostate cancer in a large Swedish study (Lindstrom et al. 2008).

The Y chromosome contains the largest non-recombining region in the human genome, spanning almost the entire length of the chromosome. This region is called the non-recombining Y (NRY) or the male-specific Y (MSY) (Rozen et al. 2003). In the absence of recombination, the NRY passes mostly unchanged from father to son and observed mutations reflect the evolutionary history of the Y chromosome. Binary markers can be used to classify Y chromosomes into haplogroups organized by a phylogenetic tree. A first generation phylogeny of the tree was published in 2002 by the Y Chromosome Consortium (2002) and further revised in 2008 (Karafet et al. 2008). The Y chromosome tree now consists of over 300 haplogroups organized into 20 major groups or clades (Karafet et al. 2008).

Multiple lines of evidence support a possible role for genes on the Y chromosome in prostate cancer etiology. Loss of the Y chromosome is one of the most frequent cytogenetic change seen in prostate tumors and may be an early event in tumorigenesis (Brothman et al. 1999; Jordan et al. 2001). In support of the previous assertion, chromosome transfer studies indicate that the human Y chromosome suppresses tumorigenicity of human prostate cell lines in vivo implying that it may harbor gene(s) with tumor suppressor function (Vijayakumar et al. 2005). Based on the essential role of the Y chromosome in secondary sexual differentiation and its potential role in disease pathogenesis, particularly related to the secondary sex organs, we explored this genomic region to investigate whether germline variation on this chromosome plays a role in prostate cancer risk.

## Results

**Fig. 1:**
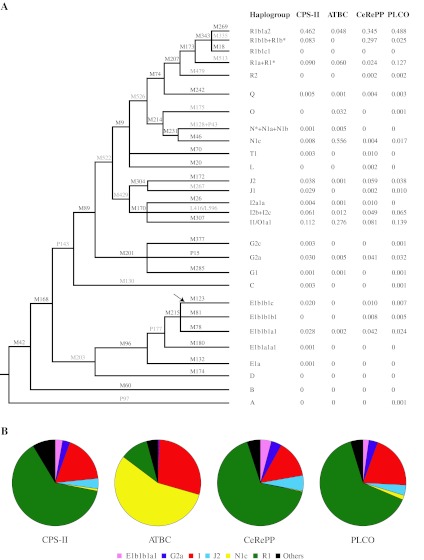
Chromosome Y haplogroup tree and frequency distribution in control subjects of European ancestry in Stage I. **a** Chromosome Y tree showing genotyped markers in *black* and those not genotyped in *light grey*. Haplogroup names are according to the International Society of Genetic Genealogy (ISOGG) 2011 update. The *arrow* points to the mutational event which gave rise to the E1b1b1c haplogroup. Stage I studies are the following: *CPS-II* American Cancer Society Cancer Prevention Study II, *ATBC* Alpha-Tocopherol, Beta-Carotene Cancer Prevention Study, *CeRePP* Centre de Recherche pour les Pathologies Prostatiques, and *PLCO* Prostate, Lung Colorectal and Ovarian Cancer Screening Trial. **b** The *circle plots* show frequencies for haplogroups with a derived frequency of 5% or higher in *different colors* for each Stage I cohort (remaining haplogroups are combined in one group shown in *black*)

We analyzed 7,810 men from the Cancer Genetic Markers of Susceptibility (CGEMS) scan in stage I of this study. Of the 34 chromosome Y markers genotyped, 26 were observed in our sample (8 markers were monomorphic). With such a sample size, we were able to accurately characterize and estimate the Y chromosome frequency distribution in populations of European ancestry for 28 haplogroups including three combined groups (R1b1b + R1b*, R1a + R1* and I2b + I2c) as the leaf nodes of the NRY tree (Fig. 1a). Stage I had 41, 76 and 95% power to detect an association with an odds ratio of 1.3 and a MAF of 0.02, 0.05 and 0.10, respectively (assuming prostate cancer prevalence of 1.5067% and alpha of 0.05) (http://seer.cancer.gov/csr/1975_2007/).

### Stage I association analysis

After genotyping quality control based on completion rates and concordance analysis, a total of 3,995 prostate cancer cases and 3,815 control subjects from four studies were used in the analysis (1994; Calle et al. 2002; Gohagan et al. 2000; Valeri et al. 2003). This included 1,531 men diagnosed with non-aggressive prostate cancer (Gleason score <7 and disease stage <III) and 2,142 men diagnosed with aggressive prostate cancer (Gleason score ≥7 or stage ≥III).

Of the 26 haplogroup markers analyzed, one was significantly associated with overall prostate cancer at a nominal *P* value threshold of *P* ≤ 0.05 (Table 1). This was haplogroup E1b1b1c (locus M123, formerly named haplogroup E3b1c) (*P* = 0.012, allelic odds ratio (OR) 0.51; 95% confidence interval 0.30–0.87), a rare haplogroup with a 1.1% frequency in control subjects in our sample set. When the analysis was performed according to degree of differentiation and severity of prostate cancer, this haplogroup was significantly associated with non-aggressive prostate cancer (*P* = 0.017, allelic OR 0.33; 95% confidence interval 0.13–0.86) but not with aggressive prostate cancer (*P* = 0.091, allelic OR 0.59; 95% confidence interval 0.32–1.09). However, the difference between the two case groups was not significant (*P* = 0.48).

**Table 1:** Association of chromosome Y variants with risk of prostate cancer (Stage I)

Haplogroup^a^	Locus^b^	rs number^c^	Location^d^	Alleles^e^	All cases				Nonaggressive cases				Aggressive cases			
					Subjects^f^	MAF^g^	*P* ^h^	OR	Subjects^f,i^	MAF^g^	*P* ^h^	OR	Subjects^f^	MAF^g^	*P* ^h^	OR
E	M96	rs9306841	20238386	C|G	3739|3903	0.042|0.043	0.638	1.06 (0.84–1.32)	3233|1476	0.039|0.029	0.188	0.78 (0.54–1.13)	3739|2111	0.042|0.056	0.242	1.16 (0.90–1.50)
E1b1b	M215	rs2032654	13977218	T|C	3807|3983	0.042|0.042	0.746	1.04 (0.83–1.30)	3298|1527	0.039|0.029	0.196	0.79 (0.55–1.13)	3807|2134	0.042|0.055	0.303	1.14 (0.89–1.48)
E1b1b1a1	M78	na*	20352691	G|A	3753|3948	0.023|0.029	0.112	1.26 (0.95–1.67)	3247|1512	0.021|0.022	0.725	1.08 (0.70–1.67)	3753|2116	0.023|0.036	0.082	1.33 (0.96–1.83)
E1b1b1b1	M81	rs2032640	20351960	G|A	3243|3372	0.002|0.001	0.079	0.27 (0.06–1.29)	2738|1223	0.001|0.000	NA	NA	3243|1945	0.002|0.001	0.192	0.36 (0.07–1.77)
E1b1b1c	M123	na*	20223974	C|T	3814|3992	0.011|0.005	0.012	0.51 (0.30–0.87)	3306|1531	0.011|0.003	0.017	0.33 (0.13–0.86)	3814|2140	0.011|0.007	0.091	0.59 (0.32–1.09)
F	M89	rs2032652	20376701	A|G	3813|3994	0.043|0.044	0.641	1.05 (0.85–1.31)	3304|1531	0.040|0.031	0.267	0.82 (0.58–1.16)	3813|2141	0.043|0.057	0.255	1.16 (0.90–1.49)
G	M201	rs2032636	13536923	G|T	3804|3987	0.029|0.026	0.528	0.92 (0.70–1.20)	3296|1529	0.027|0.026	0.995	1.00 (0.68–1.48)	3804|2136	0.029|0.029	0.388	0.87 (0.62–1.20)
G1	M285	rs13447378	21151128	G|C	3807|3990	0.001|0.001	0.973	1.02 (0.26–4.11)	3300|1528	0.001|0.002	0.440	1.86 (0.38–9.12)	3807|2140	0.001|0.000	NA	NA
G2a	P15	na*	21653414	G|A	3791|3948	0.026|0.023	0.328	0.87 (0.65–1.16)	3302|1529	0.024|0.022	0.714	0.92 (0.61–1.41)	3791|2098	0.026|0.025	0.310	0.84 (0.59–1.18)
G2c	M377	na*	13536827	A|G	3750|3950	0.002|0.002	0.410	1.54 (0.55–4.35)	3241|1508	0.002|0.002	0.843	1.16 (0.28–4.85)	3750|2120	0.002|0.002	0.474	1.56 (0.46–5.29)
I	M170	rs2032597	13357186	A|C	3811|3990	0.203|0.221	0.059	1.11 (1.00–1.24)	3302|1529	0.213|0.247	0.042	1.17 (1.01–1.35)	3811|2139	0.203|0.204	0.058	1.14 (1.00–1.31)
I1/O1a1	M307	rs13447354	21160339	G|A	3804|3988	0.150|0.154	0.648	1.03 (0.91–1.17)	3298|1525	0.161|0.176	0.737	1.03 (0.87–1.22)	3804|2141	0.150|0.136	0.249	1.10 (0.94–1.29)
I2a1a	M26	rs2032629	20325209	C|T	3719|3875	0.003|0.002	0.298	0.62 (0.25–1.53)	3215|1453	0.002|0.003	0.537	1.51 (0.41–5.58)	3719|2107	0.003|0.002	0.117	0.41 (0.13–1.30)
J	M304	rs13447352	21159241	A|C	3742|3922	0.048|0.048	0.635	1.05 (0.85–1.31)	3234|1492	0.046|0.035	0.302	0.84 (0.60–1.17)	3742|2116	0.048|0.059	0.379	1.12 (0.87–1.43)
J2	M172	rs2032604	13479028	T|G	3780|3958	0.033|0.033	0.657	1.06 (0.82–1.37)	3272|1516	0.028|0.022	0.697	0.92 (0.61–1.39)	3780|2124	0.033|0.043	0.493	1.11 (0.83–1.47)
K	M9	rs3900	20189645	C|G	3799|3975	0.322|0.340	0.055	1.10 (1.00–1.21)	3294|1525	0.324|0.338	0.299	1.07 (0.94–1.22)	3799|2129	0.322|0.348	0.021	1.15 (1.02–1.29)
T1	M70	rs2032672	20353269	T|G	3708|3904	0.003|0.003	0.922	1.04 (0.44–2.47)	3202|1492	0.002|0.001	0.315	2.92 (0.33–25.65)	3708|2101	0.003|0.004	0.882	0.93 (0.37–2.32)
N	M231	rs9341278	13979118	C|T	3769|3946	0.139|0.136	0.664	0.96 (0.80–1.15)	3262|1505	0.160|0.189	0.565	0.94 (0.76–1.17)	3769|2122	0.139|0.062	0.076	0.78 (0.60–1.03)
N1c	M46/Tat	rs34442126	13431977	T|C	3809|3992	0.137|0.134	0.604	0.95 (0.80–1.14)	3302|1530	0.157|0.185	0.514	0.93 (0.75–1.16)	3809|2140	0.137|0.062	0.058	0.77 (0.59–1.01)
NO	M214	rs2032674	13981319	A|G	3805|3977	0.139|0.135	0.676	0.96 (0.80–1.15)	3297|1523	0.159|0.186	0.578	0.94 (0.76–1.17)	3805|2135	0.139|0.062	0.058	0.77 (0.59–1.01)
P	M74	rs2032635	20349155	C|T	3547|3766	0.484|0.494	0.272	1.06 (0.96–1.18)	3047|1418	0.491|0.455	0.959	1.00 (0.86–1.16)	3547|2041	0.484|0.427	0.282	1.07 (0.95–1.21)
Q	M242	rs8179021	13527976	C|T	3719|3856	0.004|0.004	0.886	1.05 (0.51–2.19)	3229|1487	0.004|0.003	0.842	0.89 (0.27–2.88)	3719|2058	0.004|0.005	0.353	1.47 (0.65–3.35)
R	M207	rs2032658	14091377	T|C	3745|3908	0.486|0.499	0.088	1.09 (0.99–1.21)	3238|1497	0.490|0.453	0.663	0.97 (0.84–1.12)	3745|2098	0.486|0.436	0.094	1.11 (0.98–1.25)
R1	M173	rs2032624	13535818	A|C	3691|3876	0.477|0.487	0.144	1.08 (0.97–1.19)	3189|1481	0.500|0.467	0.783	0.98 (0.85–1.13)	3691|2076	0.477|0.423	0.160	1.09 (0.97–1.23)
R1b	M343	rs9786184	2947824	C|A	3805|3976	0.441|0.428	0.114	0.92 (0.84–1.02)	3297|1525	0.410|0.371	0.673	0.97 (0.84–1.12)	3805|2131	0.441|0.495	0.193	0.92 (0.82–1.04)
R1b1a2	M269	rs9786153	21148755	T|C	3489|3634	0.437|0.421	0.054	0.90 (0.81–1.00)	3029|1386	0.405|0.365	0.719	0.97 (0.84–1.13)	3489|1959	0.437|0.491	0.093	0.90 (0.79–1.02)

Results from the unconditional logistic regression of the genotypes generated in a total of 3,995 individuals with prostate cancer and 3815 control subjects are shown. The analysis was adjusted for age in 10-year categories, study and one principal component of population stratification

na* no rs number, *OR* odds ratio, *CI* 95% confidence interval

^a^Haplogroup being tested based on the Y Chromosome Consortium and ISOGG 2011 nomenclature

^b^ChrY locus/marker name

^c^NCBI dbSNP identifier

^d^NCBI human genome build 36 location

^e^Ancestral allele, derived allele

^f^Controls, cases

^g^Minor allele frequency in control and case participants

^h^Score test (1*df*)

^i^For the nonaggressive model, subjects in two categories (“FPCC” and “age <50”) could not be used because we there are no case subjects in either category. Therefore, the total number of controls is smaller than in the overall and aggressive models

Based on the Y chromosome haplogroup tree structure (see “Methods” section and Fig. 1a), we were able to test three additional haplgroups (J1, IJ, IJK) for which markers were not directly genotyped. These three haplogroups did not significantly associate with overall risk of prostate cancer (data not shown) (Table 1).

### Population substructure of E1b1b1c carriers

**Fig. 2:**
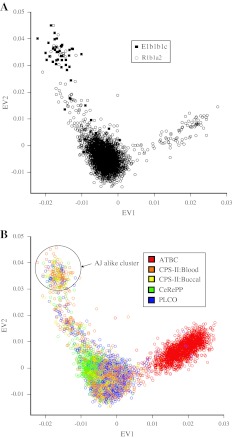
Population substructure analysis by principal component analysis and comparison to CGEMS prostate cancer GWAS. **a** shows the distribution of the first two principal components, EV1 and EV2, for carriers of E1b1b1c (*filled squares*) and R1b1a2 (*open circles*) haplogroups in Stage I. *Circles* and *squares* denote eigenvalues from PCA analysis for each individual. The distribution of EV1 and EV2 for all Stage I subjects is shown in **b**. Studies are designed by *different colors*. *CPS-II Blood* blood derived DNA samples were used for genotyping, *CPS-II Buccal* buccal derived DNA samples were used for genotyping. DNA samples from ATBC, CeRePP and PLCO were all derived from blood. Individuals of inferred Ashkenazi Jewish ancestry are *circled*. PCA results were performed by EIGENSTRAT in CGEMS prostate cancer GWAS (Thomas et al. 2008; Yeager et al. 2007, 2009)

We assessed the population substructure for E1b1b1c haplogroup carriers using principal components analysis (PCA) from the initial CGEMS GWAS dataset (Thomas et al. 2008; Yeager et al. 2007, 2009). In addition we evaluated one common haplogroup, R1b1a2 (43.7% frequency in control subjects from stage I) as it had the second lowest *P* value in stage I (*P* = 0.054). Carriers of the E1b1b1c haplogroup showed a distinct distribution of the first and second eigenvectors (EV1 and EV2) in this analysis that separates them from the majority of the European ancestry subjects, i.e. negative values for EV1 and positive values for EV2 (Fig. 2a). Conversely, the population substructure of R1b1a2 haplogroup carriers was similar to that of the majority of subjects in our study, implying Northwestern European ancestry. We compared the population substructure pattern of E1b1b1c haplogroup carriers to the large number of individuals in the initial CGEMS prostate cancer scan (Thomas et al. 2008; Yeager et al. 2007, 2009) and a GWAS of breast cancer in families of Ashkenazi Jewish descent (Gold et al. 2008) and noted strong clustering with a group of individuals of self-reported Ashkenazi Jewish descent with similar values for EV1 and EV2 (Fig. 2b). The majority of E1b1b1c haplogroup carriers in our study (37 out of 62) were Ashkenazi Jewish alike by this comparison. Therefore, the frequency of E1b1b1c in inferred Ashkenazi Jewish individuals in our study was estimated to be approximately 15% (37/240). A similar number has been reported in men of Jewish ancestry (Hammer et al. 2009). These findings prompted a replication phase in sample sets of both European and Ashkenazi Jewish ancestry.

### Limited evidence for association to prostate cancer in Stage II analysis

We attempted replication of the E1b1b1c and R1b1a2 haplogroups in three prostate cancer cohort studies of European ancestry from the continental USA, the Physicians’ Health Study (PHS) (Ma et al. 2008), the Health Professionals Follow-up Study (HPFS) (Chen et al. 2005), the Agricultural Health Study (AHS) (Alavanja et al. 1996); and in two case–control studies of Ashkenazi Jewish ancestry collected in the USA, from the Albert Einstein College of Medicine (Einstein) (Agalliu et al. 2009) and the Memorial Sloan Kettering Cancer Center (MSKCC) (Gallagher et al. 2010). The three European ancestry studies included a total of 1,272 prostate cancer cases and 1,932 control subjects; the two Ashkenazi Jewish ancestry studies included a total of 1,686 prostate cancer cases and 1,597 control subjects. Neither haplogroup was significantly associated with overall prostate cancer risk at a nominal *P* value in any study (Table 2) nor was a meta-analysis of the combined studies significant (*P*_meta_ = 0.078 for E1b1b1c and *P*_meta_ = 0.36 for R1b1a2). For non-aggressive prostate cancer, the E1b1b1c haplogroup was significantly associated at a nominal *P* value in the Einstein study only (*P* = 0.024, allelic OR 0.66; 95% confidence interval 0.45–0.95). However, a meta-analysis of non-aggressive cases in all stage II studies was nominally significant (*P*_meta_ = 0.025, allelic OR 0.68; 95% confidence interval 0.49–0.95). No other significant associations were noted.

**Table 2:** Replication analysis of notable chromosome Y signals in prostate cancer studies of Ashkenazi and European descent (Stage II)

Study	All cases				Nonaggressive cases				Aggressive cases			
	Subjects^c^	MAF^d^	*P* ^e^	OR	Subjects^c^	MAF^d^	*P* ^e^	OR	Subjects^c^	MAF^d^	*P* ^e^	OR
E1b1b1c^a^ (*M123* ^b^)												
Einstein	1214|928	0.138|0.117	0.146	0.82 (0.63–1.07)	1214|413	0.138|0.097	0.024	0.66 (0.45–0.95)	1214|456	0.138|0.134	0.693	0.94 (0.68–1.29)
MSKCC	375|750	0.128|0.123	0.608	0.87 (0.52–1.46)	375|212	0.128|0.090	0.956	0.96 (0.47–1.94)	375|362	0.128|0.146	0.954	0.98 (0.53–1.83)
PHS	489|471	0.029|0.021	0.455	0.73 (0.32–1.67)	489|194	0.029|0.015	0.379	0.57 (0.16–2.02)	489|167	0.029|0.024	0.780	0.85 (0.28–2.63)
HPFS	244|215	0.033|0.023	0.540	0.70 (0.23–2.18)	244|138	0.033|0.036	0.848	1.12 (0.36–3.49)	244|44	0.033|0.000	0.210	0.00 (0.00–1.77)
R1b1a^2^ (*M269* ^b^)												
Einstein	1210|912	0.095|0.115	0.121	1.25 (0.94–1.67)	1210|408	0.095|0.120	0.147	1.30 (0.91–1.87)	1210|446	0.095|0.108	0.390	1.17 (0.82–1.69)
MSKCC	373|739	0.110|0.118	0.936	1.02 (0.61–1.71	373|206	0.110|0.121	0.981	0.99 (0.51–1.93)	373|358	0.110|0.126	0.841	1.06(0.59–1.92)
PHS	482|463	0.500|0.531	0.321	1.14 (0.88–1.47)	482|190	0.500|0.553	0.238	1.23 (0.87–1.72)	482|164	0.500|0.537	0.430	1.15 (0.81–1.65)
AHS	1159|571	0.589|0.578	0.662	0.96 (0.78–117)	1159|326	0.589|0.558	0.320	0.88(0.66–1.09)	1159|80	0.589|0.612	0.703	1.09 (0.69–1.74)

Results from the unconditional logistic regression of the genotypes generated in Stage II are shown. The analysis was adjusted for age in 10-year categories

*OR* odds ratio, *CI* 95% confidence interval

^a^Haplogroup being tested based on the Y Chromosome Consortium and ISOGG 2011 nomenclature

^b^ChrY locus/marker name

^c^Controls, cases

^d^Minor allele frequency in control and case participants

^e^Score test (1*df*) for all scenarios except for aggressive cases from HPFS which is based on a Fisher exact test (1*df*)

A meta-analysis of stage I and II results for the E1b1b1c haplogroup revealed a nominally significant association with risk of prostate cancer overall (*P*_meta_ = 0.010; allelic OR = 0.77; 95% confidence interval 0.62–0.94) and with risk of non-aggressive prostate cancer (*P*_meta_ = 0.0077; allelic OR = 0.67; 95% confidence interval 0.50–0.90) but not with risk of aggressive prostate cancer (*P*_meta_ = 0.28). In Stage II, we had 60% power to detect a variant with 13% MAF and an OR of 0.8 in the Ashkenazi Jewish sample set, but only 25% power to detect a variant with 3% MAF and an OR of 0.7 in the European American sample set.

### Haplogroup frequency and population distribution

Y chromosome haplogroup frequency distribution in controls from each of the four study populations from phase I was summarized and compared in Fig. 1b. Two of the studies, namely CPS-II and PLCO, include subjects from continental USA. Their haplogroup frequencies are very similar with an average difference of 0.8% and a maximum difference of 5.8% for the combined category of haplogroups R1b1b + R1b*. The CeRePP study, conducted in France, is relatively similar to the US studies with an average haplogroup frequency difference of 2.2%, and a maximum difference of 24.3% for the combined group of R1b1b + R1b*. The greatest difference in frequency was seen for ATBC, a Finnish study, with an average haplogroup frequency difference of 5.2% and a maximum difference of 54.4%. This stems from a very high frequency of haplogroup N1c in this study (55.6%), while it is infrequent in the other three studies from the US and France (0.8% in CPS-II, 1.7% in PLCO and 0.4% in CeRePP). Second, R1b, the most frequent haplogroup overall, is seen in over 50% of subjects in PLCO, CPS-II and CeRePP but only in 4.8% of Finnish subjects. The third largest difference was noted for haplogroup I1 which was more common in Finns at 27.6%, as compared to 13.9% in PLCO, 11.2% in CPS-II and only 8.1% in CeRePP.

Haplogroup E1b was observed at low frequencies in all studies and its sub lineage E1b1b1c was seen in approximately 1–2% of subjects from the two US studies (PLCO and CPS-II) and the French study (CeRePP), whereas it was absent from the Finnish study (ATBC). Other haplogroups were absent or rare in the four studies.

## Discussion

In this study, we explored the role of germline Y chromosome variation in prostate cancer risk. Previous studies have not analyzed such a large sample size with as many markers in individuals of European ancestry. Because of the threshold for MAF chosen for this study (≥1%), we had limited capacity to detect risk variants with low to medium frequency and effect sizes. Prostate cancer GWAS to date have used arrays with limited coverage on the Y chromosome. As an example, in CGEMS, of the approximately 500,000 SNPs genotyped in stage I, only ten Y chromosome markers passed quality control assessment and were included in the primary analysis; this limited set of variants on the Y chromosome included only four that mark chromosome Y haplogroups (Thomas et al. 2008; Yeager et al. 2007, 2009). Other published prostate cancer GWAS studies have reported on a similar fraction of Y variants (Amundadottir et al. 2006; Chung and Chanock 2011; Eeles et al. 2008, 2009; Gudmundsson et al. 2007a, b, 2008, 2009; Haiman et al. 2007; Kote-Jarai et al. 2011; Schumacher et al. 2011; Takata et al. 2010; Thomas et al. 2008; Yeager et al. 2007, 2009).

One haplogroup of interest was noted in phase I of our study; the E1b1b1c haplogroup was nominally significant in the overall prostate cancer and non-aggressive prostate cancer groups. The marker that denotes this haplogroup is located in the last intron of the taxilin gamma 2 pseudogene (*TXLNG2P)* on chromosome Yq11.222. This haplogroup was analyzed in a second phase using replication studies of European and Ashkenazi Jewish ancestry along with a more common haplogroup, R1b1a2. Neither haplogroup was significantly associated with overall prostate cancer risk in stage II. A meta-analysis of stage I and stage II results yielded a *P* value of 0.010 for the E1b1b1c haplogroup. Although nominally significant, this *P* value is unremarkable in comparison with the rigorous threshold required for significance in GWAS studies (Wellcome Trust Case Control Consortium 2007), suggesting that further studies are required to establish this association. Although our analysis does not provide strong evidence for a relationship between variation in the Y chromosome and prostate cancer, it can be argued that the appropriate statistical threshold to be applied to a study of approximately 30 markers should not be as stringent as a GWAS threshold. However, the probability of false-positive findings is high, even in a study of our size and power (Wacholder et al. 2004) especially in the first stage where E1b1b1c haplogroup frequency was very low. In addition, we cannot exclude a chance finding due to population stratification.

Our study represents the largest analysis to date of a possible association between Y chromosome variants and prostate cancer. The role of germline variation on the Y chromosome had been assessed previously, but with limited sample and/or marker sets. One of the most complete studies published was conducted within the MEC (Paracchini et al. 2003). Four ethnic groups with a total of 930 cases and 1,208 control subjects were included. One of the 41 haplogroups observed in the study was significantly associated with prostate cancer risk in Japanese men with a *P* value of 0.02 (Paracchini et al. 2003). Despite the large overall sample set in this study, each ethnic group only consisted of approximately 100–150 case–control pairs, limiting power considerably. No haplogroups were significantly associated with prostate cancer risk in a small Korean study that assessed 14 markers in approximately 106 cases and 110 control subjects, including the haplogroup reported in the MEC study (Kim et al. 2007). Lack of an association between Y haplogroups and prostate cancer was also reported in a Swedish study assessing five ChrY markers in 1,452 cases and 779 control subjects of N-European background (Lindstrom et al. 2008). Our results appear to confirm an overall lack of importance for germline variants on the Y chromosome and prostate cancer risk.

Frequencies of Y chromosome haplogroups vary considerably between different geographical regions and ethnic groups, and have turned out to be informative in studies of human evolution and migration. In Europe, marked differences in haplogroup frequencies are observed between countries in Northeastern, Northwestern, Southwestern, Southeast and Central Europe (Wiik 2008). In addition, the Ashkenazi Jewish community has a specific pattern that is reminiscent of non-Ashkenazi Jewish communities in the Near East (Behar et al. 2004). We observed a different distribution of major haplogroups in subjects of Northwestern European ancestry (represented by the majority of subjects from the US in PLCO and CPS-II), Northeastern European ancestry (represented by Finnish subjects in ATBC) and Western/Central European ancestry (represented by French subjects in CeRePP). Haplogroups in the US and French studies can mostly be accounted for by the R and I haplogroup clans with a combined frequency of 81–85%; R1b1a2 and I1 were the most common sub branches. The R1 haplogroup clan originated in Eurasia and migrated into Europe where it divided into two subgroups, R1a (common in Eastern Europe) and R1b (common in Western Europe) (Wiik 2008). R1b1a2 shows an East to West gradient in Europe and is very common in Spain, France, UK and Ireland (Balaresque et al. 2010). Haplogroup clan I1 appears to have originated in the Balkans and migrated north throughout Europe (Wiik 2008). It is most common in Scandinavia and Northwestern Europe and gradually decreases in Central and Southern Europe (Wiik 2008). Finnish subjects were strikingly different from the other three studies with a preponderance of N1c (56%) and I1 (28%) haplogroups and few R1b carriers. The N1c haplogroup is thought to have an Eastern or Central Asian origin and probably reached Eastern Europe via expansion through Siberia (Rootsi et al. 2007). The frequency of this haplogroup in Finland has been reported to be 58% (Wiik 2008).

Genotypes in stage II confirmed the scarcity of E1b1b1c in subjects of European ancestry (1–2%) and revealed a higher frequency in the two Ashkenazi Jewish studies (13–14%), in line with previous reports (Hammer et al. 2009) indicating similar Y chromosome haplogroup frequencies in men of Ashkenazi Jewish descent living in the US and those from Jewish communities in the Middle East. E1b1b1c may have arisen in Northeastern Africa, and migrated through the Levantine corridor to the Near East and Europe (Semino et al. 2004). In a similar manner, haplogroup R1b1a2 was seen in 50–59% of the subjects in different European American studies but only 10–11% in the two Ashkenazi Jewish studies.

In conclusion, we found limited evidence for an association between Y chromosome haplogroups and risk of prostate cancer in populations of European and Ashkenazi Jewish ancestry using a large sample set close to 4,000 case–control pairs in Stage I and 2,300 case–control pairs in Stage II. Weak but consistent evidence for a protective effect for haplogroup E1b1b1c was seen in all studies with a nominally significant meta-analysis, thus, calling for additional replication efforts for this haplogroup in populations of Ashkenazi Jewish and European ancestry. The different frequencies seen in subjects from the four stage I studies may limit power to detect true associations for some branches of the Y haplogroup tree. Furthermore, correcting for population substructure based on autosomal SNPs may not be optimal, as Y chromosome inheritance only reflects male lineages that may have somewhat different characteristics throughout human history and population migration as compared to that of females. Although we cannot exclude a role for all chromosome Y haplogroups in prostate cancer etiology, our study has good power to detect common alleles with relatively large effects. Smaller or population specific effects for the haplgroups tested here, or for other haplogroups, could exist and should be studied by testing comprehensive sets of chromosome Y haplogroup markers in additional studies.

## Materials and methods

### Study population

Stage I of this study included 3,995 men diagnosed with adenocarcinoma of the prostate and 3,815 control subjects from three case–control studies nested within cohorts and one hospital based case–control study, previously analyzed in stages I and II of the Cancer Genetics Markers of Susceptibility study (CGEMS). Study details have been published previously (Thomas et al. 2008; Yeager et al. 2007, 2009).The cohort studies were: the Prostate, Lung Colorectal and Ovarian Cancer Screening Trial (PLCO, subjects from continental USA) (Gohagan et al. 2000); the American Cancer Society Cancer Prevention Study II (CPS-II, from continental USA) (Calle et al. 2002) and the Alpha-Tocopherol, Beta-Carotene Cancer Prevention Study (ATBC, from Finland) (1994). The case–control study was the French Prostate Case–Control Study (CeRePP, Centre de Recherche pour les Pathologies Prostatiques, from France) (Valeri et al. 2003). The number of subjects included from each study is shown in Supplemental Table 1a. We incorporated prostate cancer stage and grade at diagnosis to distinguish between non-aggressive (Gleason score <7 and disease stage <III, *n* = 1,531) and aggressive prostate cancer (Gleason score ≥7 and/or disease stage ≥III, *n* = 2,141) as defined in CGEMS (Thomas et al. 2008).

Stage II included 471 prostate cancer cases and 490 control subjects of European descent from the Physicians’ Health Study (PHS, from continental USA) (Ma et al. 2008); 215 prostate cancer cases and 244 control subjects of European descent from the Health Professionals Follow-up Study (HPFS, from continental USA) (Chen et al. 2005); 586 prostate cancer cases and 1198 control subjects of European descent from the Agricultural Health Study (AHS, from NC and IA) (Alavanja et al. 1996); 933 prostate cancer cases and 1,221 control subjects of Ashkenazic descent collected by the Albert Einstein College of Medicine (Einstein, majority recruited from NY, FL, CA or NJ, USA) (Agalliu et al. 2009); and 753 prostate cancer cases and 376 male control subjects of Ashkenazic descent collected at the Memorial Sloan Kettering Cancer Center (MSKCC, from Northeast USA) (Gallagher et al. 2010). Prostate cancer stage and grade at diagnosis were included to distinguish between non-aggressive (Gleason score <7 AND disease stage <III, *n* = 194 for PHS; *n* = 172 for HPFS, *n* = 338 for AHS; *n* = 416 for Einstein and *n* = 212 for MSKCC) and aggressive prostate cancer (Gleason score ≥7 OR disease stage ≥III, *n* = 167 for PHS; *n* = 80 for HPFS, *n* = 85 for AHS; *n* = 457 for Einstein and *n* = 364 for MSKCC).

The study protocols for each study were approved by the Institutional Review Board of each corresponding institution, and written informed consent was obtained from all study participants.

### Marker selection and genotyping

Markers were selected to detect chromosome Y haplogroups with minor allele frequencies (MAF) ≥1% in populations of European descent, using data from the International Society of Genetic Genealogy (ISOGG) (http://www.isogg.org/tree/ISOGG_YDNA_SNP_Index.html) 2011 update, the Y Chromosome Consortium (http://ycc.biosci.arizona.edu/) (Karafet et al. 2008; Underhill et al. 2001) and from HapMap (http://hapmap.ncbi.nlm.nih.gov/). TaqMan custom genotyping assays (ABI, Foster City, CA, USA) were designed and optimized for 34 biallelic chromosome Y markers (32 SNPs and 2 insertion/deletion polymorphisms) based on the Y Chromosome Consortium, ISOGG and HapMap databases.

For stage I, DNA was extracted from blood samples for all studies except a subset of CPS-II where buccal cells were used for a subset of subjects (*n* = 939). After pre-genotyping quality control at the Core Genotyping Facility (CGF) of the National Cancer Institute of the National Institutes of Health, Gaithersburg, MD, USA (http://cgf.nci.nih.gov/operations/pregenotyping-qaqc.html), 34 SNPs were genotyped on 9,501 samples in stage I using TaqMan genotyping assays (ABI, Foster City, CA, USA). The average concordance for 146 duplicate samples was 99.75%. Samples were excluded based on a completion rate <80% or ≥2 heterozygous genotypes. After genotype quality control (Supplemental Table 1a), 8,157 samples remained (including 8,011 subjects of which 7,810 men (3,995 cases and 3,815 controls) had all covariates used in the association analysis). Eight markers were monomorphic in our data set (Supplemental Table 2), thus leaving 26 polymorphic markers for analysis.

For stage II, DNA was isolated from blood for all studies except for the samples from Einstein where DNA was obtained from mouthwash. A subset of DNA samples from the AHS study (*n* = 1,858) were whole genome amplified prior to genotyping using the GenomiPhi™ version 2 kit (GE Healthcare) at the Core Genotyping Facility (CGF) of the National Cancer Institute of the National Institutes of Health, Gaithersburg, MD, USA (http://cgf.nci.nih.gov/operations/wga.html). Two SNPs (M123 for haplogroup E1b1b1c and M269 for haplogroup R1b1a2) were genotyped in stage II on 6,695 samples using TaqMan genotyping assays (ABI, Foster City, CA, USA) at CGF and on 1,213 samples at MSKCC (for MSKCC samples). This included 6,487 subjects (2,958 case and 3,529 control subjects). The E1b1b1c haplogroup was genotyped in samples from Einstein, MSKCC, PHS and HPFS; the R1b1a2 haplogroup was genotyped in samples from Einstein, MSKCC, PHS and AHS. Genotype quality control was performed in a similar manner as for stage I studies (detailed in Supplemental Table 1b). Concordance rates for duplicate samples (*n* = 91) were 99.9%.

### Statistical analysis

The association between haplogroups of the Y chromosome and prostate cancer risk was examined using a logistic regression model adjusted for age, study center and first principle component previously constructed based on CGEMS genotype data (Thomas et al. 2008; Yeager et al. 2007, 2009) as it was significant in the base model, to correct for population stratification if available. All subjects were of self-described European ancestry.

The variance weighted fixed-effect meta-analysis was performed to assess the overall statistical significance of stage II studies as well as combination of stage I together with II studies. Results were not corrected for multiple testing because of the strong dependence among the markers on this chromosome. Because all the Y haplogroups map to a haplogroup evolutionary tree, each branch in the tree can be cut and thus creating a bipartition of all individuals. Individuals under each cut will have inherited the mutation incurred on that branch. The case/control imbalance could therefore be tested by comparing two groupings of subjects. This is exactly the same as testing the genotypes of individual markers. To make full use of the data, genotypes from untyped branches were imputed if possible, based on its ancestor and sibling nodes in the tree. As an example, we could infer genotypes for J1 because both J and J2 were genotyped (Fig. 1a). We searched across all the branches in the tree and tested three additional untyped haplogroups, namely J1 (M267), IJ (M429) and IJK (M522). A branch was not analyzed if there was a directly genotyped derived/ancestor branch with a difference in frequency of <0.001. For example, M96 and M203 are almost the same because frequencies of haplogroup D in all study populations were close to 0. Thus, testing of the imputed marker M203 became redundant when the directly genotyped marker M96 was already analyzed.

### Validation by sequencing

Genotypes for the two markers selected for replication (M123 for haplogroup E1b1b1c and M269 for haplogroup R1b1a2) were confirmed by sequencing in 94 subjects from the current study. They were chosen from the PLCO, CeRePP and CPS-II studies in stage I such that approximately one-third (E1b1b1c) or half (R1b1a2) carried each haplogroup. Primers were designed with the program Primer3 (http://frodo.wi.mit.edu/primer3/) and used for PCR amplification of the genomic regions containing the 2 markers (Supplemental Table 3). PCR amplifications were performed with 10 ng genomic DNA using the AmpliTaq Gold 360 master mix (ABI). The samples were cleaned using AMPure beads (Agencourt) on a Biomek FX (Beckman Coulter). After resuspending the beads in 50 μl of water, PCR products were sequenced using primers for the two markers and an ABI PRISM Big Dye Terminator version 3.1 cycle sequencing kit (Applied BioSystems, Foster City, CA, USA). Sequencing was performed on an ABI 3730 capillary sequencer (Applied Biosystems). A 100% concordance was noted for E1b1b1c and 98.9% concordance for R1b1a2.

## URLs

http://cgf.nci.nih.gov/operations/pregenotyping-qaqc.html

http://code.google.com/p/glu-genetics/

http://ycc.biosci.arizona.edu/

http://www.isogg.org/tree/ISOGG_YDNA_SNP_Index.html

http://hapmap.ncbi.nlm.nih.gov/

## Electronic supplementary material

Below is the link to the electronic supplementary material.

Supplementary material 1 (XLS 19 kb)

Supplementary material 2 (XLS 23 kb)

Supplementary material 3 (XLS 20 kb)

Supplementary material 4 (XLS 18 kb)
